# *Aggregatibacter actinomycetemcomitans* Leukotoxin: A Powerful Tool with Capacity to Cause Imbalance in the Host Inflammatory Response

**DOI:** 10.3390/toxins3030242

**Published:** 2011-03-18

**Authors:** Anders Johansson

**Affiliations:** Department of Odontology, Umea University, SE-901 87 Umea, Sweden; Email: anders.johansson@odont.umu.se; Tel.: +46-90-7856291; Fax: +46-90-139289

**Keywords:** *Aggregatibacter actinomycetemcomitans*, leukotoxin, virulence mechanisms, proinflammatory response

## Abstract

*Aggregatibacter actinomycetemcomitans* has been described as a member of the indigenous oral microbiota of humans, and is involved in the pathology of periodontitis and various non-oral infections. This bacterium selectively kills human leukocytes through expression of leukotoxin, a large pore-forming protein that belongs to the Repeat in Toxin (RTX) family. The specificity of the toxin is related to its prerequisite for a specific target cell receptor, LFA-1, which is solely expressed on leukocytes. The leukotoxin causes death of different leukocyte populations in a variety of ways. It activates a rapid release of lysosomal enzymes and MMPs from neutrophils and causes apoptosis in lymphocytes. In the monocytes/macrophages, the toxin activates caspase-1, a cysteine proteinase, which causes a proinflammatory response by the activation and secretion of IL-1β and IL-18. A specific clone (JP2) of *A. actinomycetemcomitans* with enhanced leukotoxin expression significantly correlates to disease onset in infected individuals. Taken together, the mechanisms by which this toxin kills leukocytes are closely related to the pathogenic mechanisms of inflammatory disorders, such as periodontitis. Therapeutic strategies targeting the cellular and molecular inflammatory host response in periodontal diseases might be a future treatment alternative.

## 1. *Aggregatibacter actinomycetemcomitans*

*Aggregatibacter actinomycetemcomitans* is a gram-negative bacterium that is present in the oral cavity of a large proportion of the human population [1]. The prevalence of this bacterium shows great variation depending on the geographic origin, age and life style of the examined population [2,3]. *A. actinomycetemcomitans* is part of the normal flora in many healthy individuals but is also a major etiologic agent in some aggressive forms of periodontitis [4]. Periodontitis is a chronic infectious inflammatory disease characterized by the destruction of tooth-supporting structures [5]. The contribution of bacteria to the disease progression is poorly understood, probably due to the multifactorial background of this disease. The number and composition of bacteria in the oral biofilm, as well as life style and genetic predisposition, are factors that determine the outcome of the disease activity [6,7]. The genetic diversity among different isolates of *A. actinomycetemcomitans* is great and its ability to express and release virulence factors varies [1]. The different adhesins and fimbria expressed by this bacterium have been shown to be important factors that promote colonization at the various ecological niches of the human oral cavity [4]. *A. actinomyctemcomitans* expresses two exotoxins, a cytolethal distending toxin (Cdt) and a leukotoxin. Cdt’s are expressed by a number of gram-negative bacteria and cause death of the host cells by blocking their proliferation [8]. The leukotoxin selectively affects human cells of hematopoetic origin by binding to the lymphocyte function associated receptor 1 (LFA-1) and disrupting membrane integrity [9]. Leukotoxin belongs to the Repeat in Toxin family (RTX) and shares genome organization and molecular structures with RTX proteins produced by a number of other gram-negative bacteria [10]. The expression of leukotoxin and Cdt varies among different *A. actinomycetemcomitans* isolates and high leukotoxin expression has been shown to correlate with disease while the role of Cdt is still less clear [1]. The interaction between leukotoxin and host cells will be the focus of the present paper.

## 2. Leukotoxin Production

**Figure 1:**
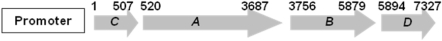
Organization of the *A. actinomycetemcomitamns* leukotoxin operon.

The *A. actinomycetemcomitans* leukotoxin operon consists of four coding genes designated *ltx*C, l*ltx*A, *ltx*B and *ltx*D and an upstream promoter [11]. *ltx*A encodes the structure of the toxin, *ltx*C for components required for posttranslational acylation of the toxin and *ltx*B and D for transport of the toxin to the bacterial outer membrane. The leukotoxin promoter is located upstream of the *ltx*C gene (Figure 1). 

**Figure 2:**
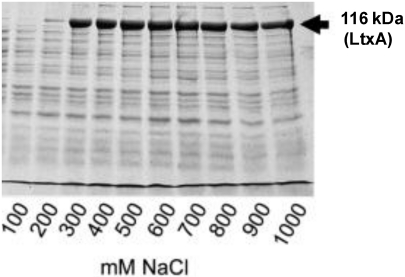
Surface extract of bacteria from a highly leukotoxic (JP2) strain of *A. actinomycetemcomitans* (HK1619) separated by SDS-PAGE and stained with Coomassie blue. The 116 kD LtxA is released from the bacterial surface in the presence of °300 mM NaCl and dominates the protein profile in these extracts [15].

There is great variation in leukotoxin expression *in vitro*, although all *A. actinomycetemcomitans* strains harbor a complete leukotoxin operon [1]. Zambon and co-workers [12] showed that A. actionomycetemcomitans isolated from periodontally diseased subjects exhibited significantly enhanced leukotoxicity compared with isolates from periodontally healthy subjects. Interestingly, certain clones of the bacterium with enhanced leukotoxin expression have been shown to have a modified promoter in the leukotoxin operon [13,14]. The cellular and molecular mechanisms in which a modified leukotoxin promoter enhances the expression of leukotoxin are not known. The most well known phenomenon is the highly leukotoxic JP2 clonal strains of *A. actinomycetemcomitans* characterized by a 530 bp deletion in the promoter of the leukotoxin operon [3]. Hypertonic NaCl extracts of bacteria from this clone separated by SDS-PAGE and stained with Coomassie, revealed a protein pattern that was dominated by a 116 kDa band shown to be the leukotoxin [15] (Figure 2). 

Presence of the JP2 clone is highly associated to aggressive forms of periodontitis and has been shown to correlate with disease onset of adolescents in Morocco [16]. This highly leukotoxic clone (JP2) has recently been reported to also colonize subjects of North-European origin confirmed by genotyping [17]. Clonal diversity analyses of JP2-like isolates have indicated that all strains of this clone have a common ancestor from Northern Africa [18]. The high accumulation of this clone in subjects of African origin has indicated a possible host tropism, but could also be a result of the strict vertical transmission pattern of this bacterium [3]. The expression of leukotoxin is also regulated by environmental factors, such as growth conditions and substrates [11]. The expression of leukotoxin by various strains of *A. actinomycetemcomitans* at the infected site of the host is still not known.

## 3. Leukotoxin Secretion

**Figure 3:**
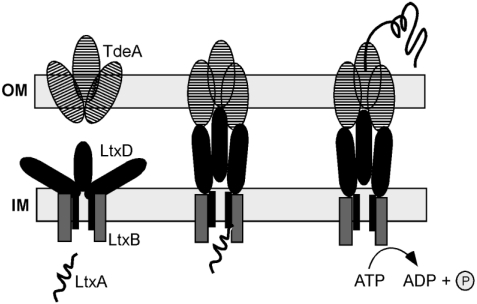
Schematic illustration of the type I secretion system required for export of the expressed *A. actinomycetemcomitamns* leukotoxin to the bacterial outer membrane (OM). IM = Inner membrane; TdeA = a TolC like protein (from Kachlany [20] with permission).

The expressed *A. actinomyctemcomitans* leukotoxin is transported to the bacterial outer membrane by a type I secretion system [11,19]. This secretion mechanism is shared with other proteins of the RTX family [10]. Three proteins of *A. actinmycetemcomitans*, LtxB, LtxD and TdeA, are reported to be required for export of the toxin to the bacterial outer membrane (Figure 3) [20]. In addition, an inner membrane protein (MorC) that affects the outer membrane structure has been shown to be necessary for efficient export of the toxin [21]. The localization of the toxin was found to be on the outside of the membrane and in membrane associated vesicles [22,23]. Whether the expressed and exported leukotoxin remains associated with the bacterial outer membrane or is secreted to the environment is a topic of controversy and the mechanisms that keep the toxin associated with the membrane are still not fully understood. A hydrophobic domain of the molecule has been suggested to mediate association with the cell membrane [24]. Ohta and co-workers [25,26] showed that leukotoxin could be released from the bacterial membrane by DNase or RNase treatment, which indicates involvement also of electrostatic forces between the negative charged nucleic acid and the positive charged leukotoxin. This was further confirmed by the observation that leukotoxin is released from the bacterial membrane and vesicles in hypertonic NaCl solutions [15] (Figure 2). In addition, presence of serum proteins also mediates release of the toxin from the bacterial outer membrane, which indicates involvement of competitive mechanisms [27]. Different culture conditions have been shown to determine the distribution of the expressed toxin between the bacterial outer membrane and the culture supernatant [28,11]. Whether leukotoxin is associated with the bacterial membrane or released to the environment at the site of infection is still not known. However, the serum mediated release of the toxin [27], as well as its highly systemic immunogenic response [29], indicates a release of the toxin from bacteria growing in an oral biofilm *in vivo*. Among the different proteins of the RTX family, *A. actinomycetemcomitans* leukotoxin differs from the other toxins by its high isoelectric point, as well as the membrane association of the expressed protein [10]. This property of LtxA further supports the importance of electrostatic forces for its association to the bacterial outer membrane.

The secreted leukotoxin has been shown to be easily inactivated by environmental proteases and superoxide radicals [30,31,32]. This degradation of the toxin molecule can be inhibited by the presence of superoxide dismutase (SOD) produced by *A. actinomycetemcomitans* and the naturally occurring protease inhibitors of human serum [31,32,33]. In 1981, McArthur and co-workers showed that the activity of leukotoxin in interaction with polymorphonuclear leukocytes (PMNs) was enhanced in the presence of human serum [34]. This phenomenon could later be explained by the protective effect of the serum protease inhibitors on leukotoxin degradation caused by lysosomal enzymes released by the affected PMNs [31].

## 4. Molecular Structure of the Leukotoxin

**Figure 4:**
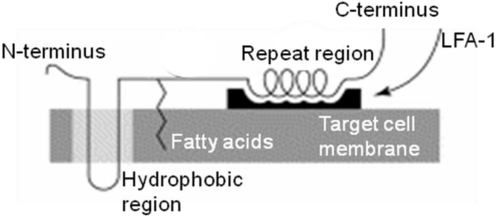
Schematic illustration of the molecular structure of the interaction between leukotoxin and the target cell membrane (from Lally [9] with permission).

The focus in this section, as well as throughout the whole manuscript, is directed to the *A. actinomycetemcomitans* leukotoxin (LtxA) if not otherwise indicated [11]. However, many of the structural and functional properties of LtxA are shared with the other proteins of the RTX family, therefore motivating a brief overview about this toxin family [10]. The RTX proteins are a highly diverse steadily growing family of toxins secreted from a number of gram negative bacterial species and with a wide range different biological activities [10]. RTX proteins fall into two categories: the hemolysins, which affect a variety of cell types, and the leukotoxins, which are cell-type- and species-specific [9]. RTX hemolysins appear to rely on electrostatic interactions to anchor the toxin on the cell membranes and the repeat region does not appear necessary for cell lysis [9]. In addition, a quite different and recently discovered division of the RTX family is the MRTX, which is a group of very large toxins that differ from all previously known RTX proteins by the molecular structure and gene cluster organization [10]. LtxA expressed by *A. actinomyctemcomitans* is a large pore-forming protein that consists of 1055 amino acids encoded by the *ltxA* gene in the leukotoxin operon [24,35]. The molecule can be divided into four regions based upon analysis of the amino acid sequence: the *N*-terminal region, the central region, the repeat region and the *C*-terminal region (Figure 4) [36]. These four regions in the molecule structure are shared among many of the bacterial proteins in the RTX family [10,37]. 

The *N*-terminal region, residues 1–408, of LtxA exhibits alternating hydrophobic and hydrophilic clusters and the pore-forming region have been suggested to be mediated by the hydrophobic clusters located between residues 175–400 [9,36,37]. The central region of the RTX proteins at residues 409–729 contains large hydrophilic domains and the two acylation sites of LtxA is located at lysine_562_ and lysine_687_[38]. The fatty acids at these positions have been shown necessary for the activity of the toxin and are suggested to contribute to the anchorage at the target cell membrane [38,39]. The repeat region of the RTX proteins consists of tandem repeats of a cassette with nine amino acids located between residues 730–900 and 14 such repeats have been identified in this region of LtxA [36,40]. The target cell receptor LFA-1 binds to the repeat region and this interaction has been shown to be responsible for the host cell specificity of LtxA [40,41]. In addition, the glycine rich repeats in this region have strong capacity to bind Ca^2+^ and presence of these cations mediates increased binding of the toxin to LtxA-sensitive LFA-1 expressing cells [42]. Finally, residues 901–1055 at the *C*-terminal end of the RTX proteins have been shown to be needed for export of the toxin to the bacterial outer membrane by interactions with secretory proteins [37]. This region of LtxA contains 20 extra basic amino acid residues in comparison with other RTX-proteins and is the reason for its high isoelectric point (9.7) [35]. Even though the four regions of LtxA described above are shared among the various toxins in the diverse family of pore forming RTX proteins, their amino acid sequence homology is limited to about 40–50%, with the highest homology between their repeat regions and the lowest between their *C*-terminal regions [35,37]. A partial denaturation of the LtxA molecule has been reported to enhance its leukotoxicity, which indicates that conformational changes interact with the activity of the toxin [43]. Some minor differences have been identified on the *ltxA* genes between different *A. actinomycetemcomitans* isolates but whether these differences interfere with leukotoxicity is not known [24,35,44]. The crystalline structure of LtxA has not yet been solved, which limits the available information about the molecular structures of the protein.

## 5. Interaction of LtxA with the Target Cell Membrane

LtxA expressed by *A. actinomycetemcomitans* exhibits a unique specificity against cells of hematopoetic origin from humans and some other primates [41]. This restricted host cell specificity suggests that the species-specific effect of LtxA is mediated through a unique receptor on the target cells and a precise region in the toxin that recognizes and interacts with the receptor [45,46]. The principal feature of this species recognition region of LtxA is thatit contains a series of 14 tandemly repeated amino acid sequences in the repeat region of the toxin [36,40].

LtxA has been shown to bind surfaces of toxin-sensitive LFA-1 expressing cells, as well as toxin-resistant cells without LFA-1 expression [42]. It has been suggested that the role of LFA-1 in LtxA mediated cell lysis is to help the protein to have a correct orientation on the target cell membrane (Figure 4) [9]. Further, the two fatty acids strengthen the anchorage of the toxin when inserted in the target cell membrane and the hydrophobic domain forms small pores in the membrane. It has been stated that low concentrations of the toxins might induce apoptosis through loss of membrane integrity caused by the small pores and that higher concentrations of the toxin allows oligomerizerization of LtxA-LFA-1 complexes on the target cell membrane, mediating a rapid and complete membrane collapse [9]. In addition, LtxA has been shown to require lipid rafts for target cytotoxicity, which also indicates the importance of a high mobility on the target cell membrane [47].

The domain of LtxA that recognizes the target cell receptor has been shown to be residues 688–941, examined by epitope mapping with monoclonal antibodies [40]. The LFA-1 molecule identified as the LtxA target cell receptor is a heterodimer consisting of the α_L_ (CD11a) and β_2_ (CD18) subunits. The residues 1–128 on human CD11a have been shown to be important for the human specificity of LtxA-induced cell lysis [46]. In addition, the extracellular region of human CD18 (residues 500–600) has been shown to be critical for conferring susceptibility to LtxA-induced cell lysis [45]. The most important ligand of LFA-1 is the intercellular adhesion molecule 1 (ICAM-1), but this interaction does not coincide with the residues identified for the LtxA binding [45,46,48]. This finding indicates that the intracellular signaling mediated by the LFA-1 ligand binding is not activated by the LtxA binding. Three different affinity states (low, intermediate, high) of LFA-1 that interfere with ligand binding have been described [49]. It is not known if these different affinity states of the LtxA receptor interfere with the interactions between LtxA and its target cells. 

## 6. Virulence Mechanisms of the Leukotoxin (LtxA)

The ability of *A. actinomycetemcomitans* extracts to cause death of leukocytes was first shown more than 30 years ago [50,51]. A protein named leukotoxin was identified as the responsible molecule for this leukotoxic effect that was restricted to human PMNs and monocytes [50,51,52]. It was later shown that leukotoxin can also affect human lymphocytes and erythrocytes from human and animal origin, however, at higher concentrations of the toxin than which lyses PMNs and monocytes [53,54].

### 6.1. Polymorphonuclear Leukocytes

**Figure 5:**
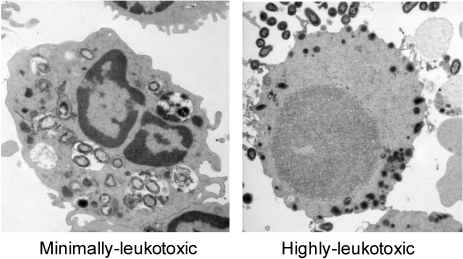
Polymorphonuclear leukocytes (PMNs) were exposed to live *A. actinomycetemcomitans* for 10 min in a ratio of 25 bacteria/PMN, in the presence of 10% human non-immune sera at 37 °C under anaerobic conditions with gentle agitation. The low leukotoxic bacteria were phogocytized and killed by the PMN (left), while the highly leukotoxic bacteria (JP2) resisted PMN phagocytosis and caused extracellular release of lysosomal components (right) [58].

PMNs are the first defense cells to be recruited in the acute phase of an inflammation, as in a periodontal infection [55]. These defense cells are often found in high numbers in the infected periodontal pocket, attracted from the peripheral circulation through chemotaxis towards a gradient of molecules released from the oral biofilm, as well as activated host cells. Although PMNs are crucial for phagocytizing and killing the bacteria, they have also been shown to release substances that mediate tissue destruction in aggressive forms of periodontitis [56]. PMNs in the periodontium have been described as a “double-edged sword”, capable of producing periodontal disease as well as protecting against such disease [57]. LtxA, as well as leukotoxic bacteria, have been shown to efficiently cause death of human PMNs, and consequently LtxA is assumed to protect *A. actinomycetemcomitans* against phagocytic killing [1]. The protection depends on the LtxA concentration of the bacterial population [58]. In a mixture of low-leukotoxic bacteria, human serum and PMNs that is agitated at 37 °C under anaerobic conditions, the bacteria are efficiently phagocytized and killed at a ratio of 25 bacteria/PMN [58]. In contrast, in the presence of highly leukotoxic (JP2-clone) bacteria under the same physiological conditions, the PMNs fail to phagocytize and kill the bacteria. Transmission electron microscopy pictures of the exposed PMNs showed a peripheral translocation of the granules in cells exposed to the highly leukotoxic bacteria (Figure 5). Further analyses of PMNs exposed to leukotoxin showed an extracellular release of proteolytic enzymes from both primary and secondary granules [30]. Moreover, the interaction between LtxA and PMNs mediates activation and release of matrix metalloproteinase 8 [59]. Taken together, these findings indicate that beyond causing death of the PMNs, LtxA also induces activation and release of proteolytic enzymes from these cells, which might contribute to the disease progression.

Whether LtxA can exist as a biologically active protein in the infected periodontal pocket has not yet been examined. The presence of serum proteins and the relatively high pH (≈8) in the pocket indicates that LtxA is released from the bacterial surface in this ecological niche [27,35]. The released toxin makes it to an easy target for inactivation by several of the components present in the periodontal pocket, such as superoxide radicals and proteinases released from the host defense cells or the colonizers of the oral subgingival biofilm [15,27,32]. In addition, it has been shown that systemic antibodies with specific reactivity against LtxA neutralize leukotoxic activity, but if these antibodies are functional in the complex ecological niche of an infected periodontal pocket is not known [29]. There are also molecules in the periodontal pocket that can protect LtxA from inactivation, such as the host serum proteinase inhibitors and bacterial SOD expressed by *A. actinomycetemcomitans* [31,32]. The great variation over time in the balance between these factors probably affects the activity of LtxA at the infected site and the burst periods observed in the pathogenesis of periodontitis might depend on such a phenomenon.

Impaired PMN function is closely associated with periodontitis and functional PMNs seems to be of certain importance when *A. actinomycetemcomitans* is present in the oral subgingival biofilm [33,55,59,60]. For instance, PMNs of subjects with Kostmann’s syndrome are immature and express truncated LL37, a cathelicidin with antibacterial effect against *A. actinomycetemcomitans* [60]. Furthermore, subjects with Papillon-Lefèvre syndrome have impaired PMN serine proteases maturation, which causes an enhanced LtxA sensitivity due to decreased capacity to degrade extracellular LtxA by the released lysosomal PMN enzymes [33].

### 6.2. Lymphocytes

The lymphocytes were initially described as LtxA resistant cells [50,51]. The first observation of LtxA susceptible cells of lymphocytic origin was by Simpson and co-workers [61] who showed that several lymphoid cell lines were killed in the presence of LtxA. In addition, LtxA was shown to suppress the function of peripheral blood lymphocytes [62]. A few years later, Mangan and co-workers [53] showed that T-cells isolated from human peripheral blood were affected by LtxA. This LtxA-induced T-cell death was a slow process compared to that which lyses human cells of myeloid origin and the cell death was shown to be induced through apoptosis [53]. It has also been shown that the human natural killer (NK) cells are affected in a similar way by LtxA as the T-cells, while the effects of LtxA on human B-cells or plasma-cells not have been specifically addressed [62]. Human lymphocytes show a great heterogeneity in regard to LtxA sensitivity and a subgroup of these cells has been shown to be lysed at approximately the same concentrations as human PMNs [63]. The lymphocytes with different LtxA sensitivity were not further characterized in this study but analyses of CD11a expression on there cell membrane showed a heterogenic distribution pattern in this cell population. The reason for the variation in LtxA sensitivity between PMNs and lymphocytes is not known. The suggested oligomerization of LtxA-LFA-1 complex and the need of lipid rafts on the target cell membrane may indicate that the composition of membrane molecules on the target cells determine the source of LtxA-induced death mechanisms [9,49]. It has been shown that low concentrations of LtxA cause apoptosis, and in higher concentrations necrosis, in cultures of a human carcinoma cell line of myeloid origin (HL-60) [64].

Cells of lymphoid origin are rare in the infected periodontal pocket but reside in high numbers in the surrounding tissues as well as in the lymph glands [65]. It has been known for >30 years that the onset of periodontitis involves a switch from a T cell lesion to one involving large numbers of B-cells and plasma cells. A shift occurs in the balance between the so-called Th1 and Th2 subsets of T-cells, with Th2 cells associated to chronic periodontitis [66]. More recently, T regulatory (Treg) and Th17 cells have been detected in periodontal tissues, indicating that these cells also are of importance in the host response and pathogenesis of periodontal disease [67]. The strong acquired systemic humoral immune response induced by LtxA indicates direct contact between this molecule and cells of lymphoid origin [29,68]. The ability of LtxA to induce apoptosis in lymphocytes might contribute to a locally impaired acquired immune response in periodontal infections. The ability of LtxA to affect also the lymphocytes indicates a possible role of this molecule in Th1/Th2/Th17 differentiation, a process that seems to be of great importance in the pathogenesis of inflammatory diseases, such as periodontitis [67].

### 6.3. Monocytes/Macrophages

It was early shown that human monocytes are sensitive targets for LtxA, and described that the sensitivity of these subsets of leukocytes was at a similar level as for human PMNs [51]. Characterization of the LtxA induced monocyte killing has described three different phases: (1) cessation of the membrane undulating folding and an accumulation of granulae in the perinuclear area; (2) abnormal membrane movement and strings of cytoplasm projecting from the cell; (3) explosive release of cytoplasmic material from the cell [52]. However, it should be taken into consideration that this study was made with a crude LtxA extract that contained a large number of other bacterial components. Rabie and co-workers [62] showed that purified LtxA caused a rapid death of human monocytes in mixtures of the toxin with peripheral blood mononuclear leukocytes (MNL).

**Figure 6:**
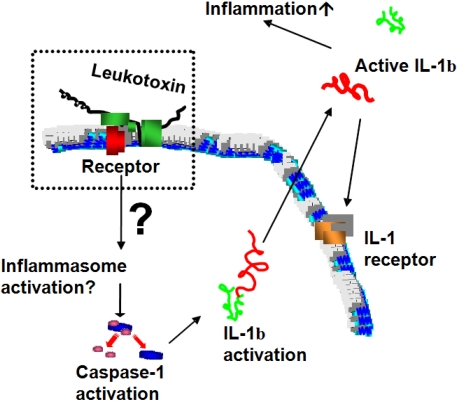
Schematic illustration of cellular mechanisms involved in LtxA-induced monocyte/macrophage death. It is suggested that LtxA adheres to the target cell membrane through binding to the LFA-1 dimer and further anchorage of the molecule by insertion of the fatty acid into the membrane lipid bilayer. The hydrophobic domain of the toxin is thought to cause small pores in the target cell membrane. Through still undefined intracellular signaling pathways, this interaction with the target cell membrane causes activation of caspase-1 and IL-1β that are secreted in a bioactive form from the affected cell.

More recently, analyses of different subsets of leukocytes separated from peripheral blood of a single donor showed that monocytes have an enhanced sensitivity to LtxA compared to PMNs and lymphocytes [63]. The LtxA-induced monocyte lysis was shown to involve activation of caspase-1, which indicates involvement of proinflammatory intracellular signaling (Figure 6). Caspase-1 is a cytosolic cysteine proteinase that specifically induces activation and secretion of the proinflammatory cytokines interleukin-1β (IL-1β) and interleukin-18 (IL-18) [69,70]. Both these proinflammatory cytokines are expressed as biologically inactive precursors and have to be cleaved by caspase-1 for activation and secretion. Caspase-1 is activated by incorporation in a cytosolic multimer complex named the inflammasome [71]. The intracellular signaling pathways involved in LtxA-induced inflammasome activation in human monocytes/macrophages have not yet been determined. A partial characterization of this process indicates involvements of K^+^ efflux and ATP-release that might activate purogenic receptors, such as the P2X_7_[72]. Caspase-1 activation is also caused by several other gram-negative pathogens, such as *Salmonella* and *Shigella* species, and it has been shown to be an important innate immune effector mechanism against intracellular bacteria [73].

The observation that LtxA induces activation of caspase-1 in human inflammatory defense cells indicates a new role of this virulence factor as a mediator of proinflammatory host response. Human macrophages (adherent blood monocytes) exposed to LtxA activate a rapid and abundant secretion of bioactive IL-1β [74]. Culture supernatants of LtxA exposed macrophages induces bone resorption in cultured mouse calvaria and the addition of monoclonal antibodies against IL-1β to the assay abolished this activation. This data indicated that bone resorption caused by culture supernatants of LtxA exposed macrophages is mainly caused by the activated and released IL-1β [74]. Moreover, exposure of human macrophages to components of gram-negative oral pathogens causes an increased accumulation of cytosolic pro-IL-1β that not is activated and released [75]. LtxA or leukotoxic *A. actinomycetemcomitans* induces cleavage and secretion of this accumulated macrophage IL-1β, a property that was abolished in A. actinomycetemomitans mutants without LtxA expression. The IL-1β secretion was activated already at a concentration of one bacteria/macrophage in interactions with bacteria from the highly leukotoxic JP2 clonal type of *A. actinomycetemcomitans* and with a similar activation by low leukotoxic bacteria at a 10-times higher concentration [75]. Taken together, these data showed that LtxA is the major component of *A. actinomycetemcomitans* for induction of activation and release of IL-1β from human macrophages and that this effect was further enhanced by priming of the macrophages with other bacterial components.

Macrophages are rare cells in a healthy periodontium but are often found in high numbers in tissues from periodontal lesions [65]. These cells are recruited to the infected site from the peripheral blood monocytes that are attracted by ICAM-1 expressing endothelial cells. The monocytes pass through the vessel wall and migrate towards a gradient of compounds in the connective tissue that are released from the oral biofilm and the activated host cells [76]. During diapedesis, the monocytes differentiate into macrophages and the inflammatory machinery is up-regulated during this process and further during the migration towards the infected site. This process involves an accumulation of proinflammatory precursor molecules, such as IL-1β and IL-18, in the migrating macrophages [77]. A secondary stimulus is needed to induce activation and release of the accumulated precursors of IL-1β and IL-18 in the primed macrophages [70]. In the case of an infection containing *A. actinomycetemcomitans*, the gradient of bioactive components in the connective tissue will contain LtxA, and the migrating macrophages will sooner or later meet concentrations of LtxA that activates secretion of these proinflammatory cytokines into the surrounding tissues. If this process is activated in the tooth supporting tissues in the vicinity to the infection, it might cause imbalance in the host inflammatory response and promote pathogenic cellular mechanisms. Initial analyses of gingival crevicular fluid indicate an association between enhanced IL-1β levels and high number of *A. actinomycetemcomitans* in the periodontal pocket [75].

The highly systemic immunogenic host response against LtxA of *A. actinomycetemcomitans* infected subjects indicates direct contact between the antigen presenting macrophages and LtxA [29,68]. The enhanced LtxA-sensitivity of human macrophages indicates that these antigen presenting cells might be affected during a primary infection with leukotoxic *A. actinomycetemcomitans*, which might cause a delayed acquired immune response.

The proinflammatory response associated with degenerative diseases is a focus of research in many different medical disciplines [70]. A variety of safe and effective anti inflammatory agents are available today and commonly used to treat many autoimmune or autoinflammatory disorders, neurodegenerative disease, or cancer. Increased knowledge of the cellular and molecular mechanisms involved in the pathogenesis of periodontitis might open up possibilities for new specific therapeutic agents and strategies in the future. The cellular and molecular targets for specific blockage of the inflammatory response to infection, as well as the possible therapeutic agents now and in the future, have recently been extensively reviewed [70]. 

### 6.4. Erythrocytes

The ability of some strains of *A. actinomycetemcomitans* to cause β-hemolysis on blood agar plates has been known for many years [78,79]. It was later determined that hemolysis of red blood cells of human and animal origin that is caused by *A. actinomycetemcomitans* involve interaction with LtxA [54]. Different strains of the bacterium with various expression of LtxA show a specific pattern when cultured on blood agar plates containing fresh horse blood. Red blood cells lack expression of the LtxA receptor LFA-1, which has been shown to be a prerequisite for LtxA-induced leukocyte lysis [40]. The cellular and molecular mechanisms for this hemolytic effect of LtxA are therefore not fully understood. The ability of LtxA to lyse red blood cells has been described in detail by Kachlany [11] in a recently published review. Whether this hemolytic property of LtxA is a virulence mechanism is still not known.

## 7. Acquired Humoral Immune Response to LtxA

The specific role of humoral immunity in periodontal disease progression has not been fully elucidated, although the induction of antibody response is suggested to be beneficial for the host in the fight against periodontal infections [80]. On the other hand, the acquired immune response against periodontal pathogens has been shown to mediate disease associated mechanisms, such as bone resorption [81,82]. It has clearly been shown that leukotoxin specific antibodies are present in the peripheral circulation of both periodontally healthy and diseased subjects [29,68]. Plasma samples from the subjects with specific immunoreactivity against LtxA have been shown to neutralize LtxA activity and contain enhanced antibody titers against whole cells of *A. actinomycetemcomitans* in comparison with samples from subjects without immunoreactivity to LtxA [68]. It has also been shown that systemic leukotoxin neutralization is correlated to the presence of this bacterium in the oral subgingival biofilm [59,83,84]. Systemic LtxA antibodies have been shown to be present in >50% of samples from adults and with a similar prevalence in periodontally healthy and periodontally diseased subjects [29,85]. Interestingly, systemic LtxA neutralizing capacity correlates to decreased risk of the incidence of stroke in women [86]. The mechanism behind this phenomenon has not yet been determined but indicates a role of LtxA in the association seen between peridontitis and cardiovascular diseases [6].

A general opinion is that the aquired humoral immune response against antigens of the oral subgingival microbiota is both local and systemic [80]. Whether the LtxA activity can be neutralized in the gingival pocket by specific antibodies is not known and there has been no report about the presence of LtxA neutralizing antibodies in the gingival crevice fluid. The strong correlation between prevalence of highly leukotoxic *A. actinomycetemcomitans* and the development of attachment loss [16] indicates a minor role of neutralizing antibodies in the infected periodontal pocket. However, it can be assumed that systemic LtxA neutralizing antibodies are an important protection against the systemic side effects that are associated with periodontitis, such as increased risk for diabetes and cardiovascular diseases [6]. It could further be speculated that the ability of LtxA to specifically affect the immune cells, in particular the antigen presenting monocytes/macrophages, causes a delayed acquired immune response in the primary *A. actinomycetemcomitans* infection.

## 8. Conclusions

The ability of LtxA to cause death of all subsets of cells with hematopoetic origin might contribute to help the bacterium to survive the host immune response and also to release compounds essential for bacterial growth. The more recent discoveries that LtxA mediates activation and release of proteolytic enzymes from PMNs and proinflammatory cytokines from monocytes/macrophages indicate a more direct role of LtxA in the pathogenesis of periodontal diseases. Unfortunately there is no animal model available for studying the virulence mechanisms of LtxA because of its specificity against defense cells of human or those of old world monkey origin. However, the strong correlation between the presence of highly leukotoxic (JP2-clone) *A. actinomycetemcomitans* and development of attachment loss in adolescents indicates an important role of LtxA in the pathogenesis of aggressive periodontitis [3]. The treatment strategies of periodontal disease are today focused on the elimination of the bacteria in the subgingval biofilm by mechanical techniques or with antibacterial agents [87]. Therapeutic agents that target the cellular and molecular inflammatory host response might be a future treatment alternative for periodontal disease. 
